# Editorial: Enhancing cancer therapy: integrating plant-derived bioactives with chemotherapy through traditional knowledge and modern advances

**DOI:** 10.3389/fphar.2026.1752854

**Published:** 2026-03-13

**Authors:** Johnson O. Oladele, Ebenezer I. O. Ajayi, Omowumi O. Adewale, Olu I. Oyewole

**Affiliations:** 1 Phytochemistry and Toxicology Unit, Royal Scientific Research Institute, Osun, Nigeria; 2 Department of Veterinary Physiology and Pharmacology, College of Veterinary Medicine and Biomedical Sciences, Texas A&M University, College Station, TX, United States; 3 Department of Biochemistry, Faculty of Basic and Applied Sciences, Osun State University, Osogbo, Nigeria; 4 The STREAMMIE Centre, Ebelola Bioenergetic Solutions, Kajola Ijesa, Nigeria

**Keywords:** cancer, chemotherapy, ethnophamacology, phytochemical, natural product, medicinal plant

## Introduction

The prevalence of cancer, which has a huge negative impact on both global health and economy, is still one of the biggest health challenges of the twenty-first century (Bizuayehu et al., 2024). Although modern targeted agents, radiotherapy, and conventional chemotherapy have greatly increased survival rates, their potency is greatly compromised by drug resistance, systemic toxicity, and a marked decline in patient quality of life. Therefore, it is crucial to develop novel therapeutic approaches that can increase the effectiveness of current treatments while reducing their adverse effects. This pressing need has led to a renewed interest in studying bioactives derived from plants. These naturally occurring substances, which have been refined over thousands of years in conventional medical systems, constitute a largely unexplored source of new chemical structures, natural products and phytochemicals with built-in multi-targeting cellular properties (Oladele et al., 2020; Kumar et al., 2023; Oladele and Okoro, 2025).

Basically, there are two reasons to the inclusion of these bioactives in contemporary cancer treatment. First, a wide range of malignant phenotypes are susceptible to the inherent antitumor activity of numerous bioactive compounds derived from plants. Secondly, and possibly more significantly, they have strong adjuvant potential that can reverse chemoresistance, making cancer cells more sensitive to these bioactive compounds, and shield healthy tissue from unintended harm. A disciplinary convergence is necessary for this paradigm shift, specifically the thorough validation of tradomedical practices via the prism of pharmacognosy, pharmacology and molecular oncology.

This important field of study is effectively summarized by this Research Topic, “Enhancing Cancer Therapy: Integrating Plant-Derived Bioactives with Chemotherapy through Traditional Knowledge and Modern Advances.” With 24 unique contributions, it collectively traces the development of the field from translational clinical research and macro-level bibliometric analyses to the clarification of new molecular mechanisms and the confirmation of synergistic combinations. The goal of this Research Topic was to facilitate the clinical adoption of these potent natural agents by bridging the gap between the historical effectiveness of botanical medicine and the accuracy and reproducibility required by modern evidence-based oncology. The wide range of research contributions to this Research Topic can be logically categorized into four related themes that cover all aspects from systemic analysis to clinical practice and molecular biology.

### Bioactives and their mechanisms

There is a growing knowledge of how specific compounds derived from plants alter basic cellular processes involved in carcinogenesis. Current methods and approaches are effectively discovering new mechanisms, going beyond cytotoxicity to target signaling cascades and cell death pathways. The review by Ye and Ju emphasizes the role natural products play in controlling ferroptosis, a type of iron-dependent programmed cell death. The review offered a comprehensive mechanistic insight how bioactives targeting ferroptosis signaling pathways altered tumor growth. Similarly, the contribution by Zhang et al. provides detailed antitumor action of Erianin in anaplastic thyroid carcinoma *via* induction of both apoptosis and pyroptosis, mediated through simultaneous inhibition of the MAPK/ERK and PI3K/Akt pathways. According to Wang et al., the study of the therapeutic effects of Miltirone on gastric cancer demonstrated its ability to significantly facilitate the chemosensitivity of cisplatin by inhibiting the PI3K/Akt signaling pathway. These results emphasize a mechanistic convergence in which bioactives consistently target hyperactive survival pathways that are essential for the growth and resistance of cancer.

Furthermore, Chen et al. reported that isoliensinine exhibited modulatory effects on cellular stress in lung adenocarcinoma by preventing APEX1-driven reactive oxygen species (ROS) generation. A separate study also discovered that dehydrodiisoeugenol inhibited the progression of breast cancer cell cycle by targeting the PLK1-p53 axis (Li et al.). According to Chen et al., the emerging mechanisms of Asiatic acid in cancer therapy, established that bioactives are constantly being reexamined with molecular rigor in addition to novel agents. Taking the synthesis efforts surrounding mangiferin derivatives with the goal of developing novel complexes and carriers as therapeutic candidates into account, Melo-Betances et al. show that the field is also addressing drug optimization. Finally, using integrated network analysis, bioinformatics, single-cell sequencing, and cell experiments, the complex mechanism of Diosgenin against gastric cancer was elucidated by Yun et al., demonstrating the ability of contemporary systems biology to validate conventional compounds.

### Synergistic combinations of bioactives in chemotherapy

The clinical potential of plant bioactives is regularly recognized in their role as adjuvant agents that enhance the efficacy of standard treatments and mitigate treatment-linked toxicities, rather than as monotherapy. Targeted experimental studies and systematic reviews present strong evidence for synergistic combinations. The effectiveness and safety of combining Chinese herbal medicine (CHM) with targeted medications and chemotherapy have been demonstrated by numerous meta-analyses. The combined use of Chinese herbal injections and EGFR-TKIs for non-small cell lung cancer (NSCLC) was examined in a systematic review and network meta-analysis (Yuan et al.), which produced strong evidence for increased clinical benefits. The comparative safety and effectiveness of eight particular Chinese herbal injections in combination with chemotherapy for breast cancer were the subject of a related study which also reported enhanced clinical response (Shi et al.). Another systematic review and network meta-analysis by Ma et al. also confirmed the widespread effectiveness of oral CHM. By further combining conventional formulas, yet another systematic review and meta-analysis investigated the therapeutic potential of modified Yukgunja-tang (Liujunzi Decoction, Rikkunshito) as a lung cancer adjuvant treatment (Kang et al.). There is general agreement from these thorough meta-analyses regarding the beneficial contribution of integrated CHM to enhancing overall treatment results.

The synergy between plant-derived compounds and conventional therapies also extends to their protective actions at the cellular level. For example, a mechanistic report of some polyphenolic compounds on cisplatin-induced ototoxicity was shown to suppress key pathways responsible for cochlear oxidative injury, hence, counteracting cisplatin toxicity. This is a further support regarding the capacity of plant bioactives to protect non-malignant tissues and, in turn, improve therapeutic selectivity. Similarly, Zhang et al. demonstrated that combining Erianin with anlotinib markedly enhances antitumor response through complementary signaling interactions. In addition, Deng et al. provided an extensive overview of the multiple synergistic functions of quercetin in immunomodulation and chemotherapy, reinforcing its increasing relevance for clinical translation.

### Translational and clinical perspectives

A number of papers in this Research Topic address the translational bridges required for clinical adoption of plant derived bioactives, specifically with regard to supportive care and quality of life improvement, i.e., the two critical Research Topic for cancer patients undergoing intensive treatment. The role of traditional herbal medicine in preventing chemotherapy-induced peripheral neuropathy (CIPN) is specifically examined in a systematic review and meta-analysis that integrates association rule analysis to identify predictive patterns (Kim et al.). Boku et al. provides human data that further elucidates the supportive role of bioactives in colorectal cancer treatment.

The way for future clinical trials is being actively paved by preclinical research. In order to build a strong case for translation, the research on Astragalus and *Dioscorea opposita* describes their preclinical studies and possible clinical use in cancer treatment (Jia et al.). The evidence base for targeted application was also consolidated by reviewing the clinical research, mechanisms, and prospects of flavonoids from *Herba patriniae* in the treatment of colorectal cancer (Zhang et al.). Additionally, a thorough review lays the groundwork for toxicology and dose-finding studies by projecting shikonin as a therapeutic candidate in female carcinomas from a preclinical perspective (Pandey et al.). Taking together, these studies offer crucial translational information that shows how botanicals cannot only combat cancer but also maintain the immunomodulatory and oxidative balance required for patients to recover and tolerate standard treatment.

### System biology, ethnopharmacology and research drifts

The crucial role that modern systems biology, research analytics, and traditional knowledge systems play in directing future discoveries is summarized in this section. The investigation of anticancer ethnomedicines for cancer treatment in Taiwan by Ko et al. serves as an example of how established ethnopharmacology forms the basis of this entire field. Modern discovery is grounded in past clinical experience owed to studies such as these, which offer the essential starting points for identifying lead compounds and complex formulations. Validating and improving these conventional leads now requires the use of contemporary research instruments. The use state-of-the-art molecular biology to explain the systemic, regulatory effects of complex natural molecules is demonstrated in the review examining how plant polysaccharides influence tumor development based on epigenetics (Li et al.). This offers a clear explanation for the systemic advantages and low toxicity frequently connected to conventional treatments.

Finally, the use of bibliometric analysis to map the global research landscape is evidence of the field’s maturation. Lei et al. mapped the research network and evolution of traditional Chinese medicine in cancer treatment *via* immune system modulation between 2015 and 2025, identifying thematic hot spots and important collaboration patterns. Another research mapped the growing importance and research focus of rhein in the treatment of tumors (Jiang et al.). Researchers can find gaps, prevent duplication, and use international cooperation to speed translational breakthroughs with the help of these bibliometric contributions, which offer insightful meta-level information.

### Future directions and perspectives

Even though the papers in this Research Topic show a lot of progress, there are still a number of important Research Topic and research gaps that need to be filled before plant-derived therapies can become widely useful. The main obstacle is the complicated pharmacokinetics and bioavailability of many bioactives, which frequently have limited systemic access, poor solubility, and fast metabolism (Wu et al., 2024). To guarantee that therapeutic doses are safely reaching the tumor sites, future research must give priority to strong pharmaceutical sciences, including innovative delivery methods like liposomal formulations and nano-carriers. The urgent need to standardize herbal formulations is inextricably linked to this. With complex botanical extracts, it can be challenging to ensure consistent, reproducible dosing, which is necessary for clinical advantage; quality control and analytical chemistry procedures need to be significantly tightened.

In order to completely describe the multi-target, poly-pharmacological effects of whole extracts and intricate TCM formulas, it is technologically necessary to integrate omics technologies (genomics, proteomics, and metabolomics) (Ryszkiewicz et al., 2025). The field will advance from identifying single-compound mechanisms to predictive modeling of compound synergy within biological systems. Integrating AI-driven drug discovery and advanced multi-target computational modeling are also vital to holistically explore advantages of plant-derived bioactives in chemotherapy (Zhai et al., 2025). The difficult process of determining the best combination ratios and therapeutic targets from the enormous chemical space provided by the plant kingdom can be sped up with AI-driven applications and processes.

To satisfy the regulatory requirements of contemporary oncology, translational efforts must change. This involves creating thorough, extensive adaptive clinical trials that use quality of life (QoL) and patient-reported outcomes (PROs) as the main endpoints in addition to the more conventional survival and response rates. More interdisciplinary cooperation is also required. From the first hypothesis to regulatory submission, oncologists, pharmacologists, medicinal chemists, ethnobotanists, and computational biologists must collaborate. We can guarantee that the cultural significance and age-old knowledge of traditional medicine systems are thoroughly verified and converted into internationally accessible, evidence-based oncologic practice by cultivating this cooperative ecosystem.

## Conclusion

This Research Topic offers a comprehensive, multifaceted view of the future of cancer treatment and has successfully culminated in 24 high-quality publications. The contributions show a potent combination of systems analysis, promising translational studies, rigorous systematic reviews, and molecular biology. The Research Topic highlights the enormous therapeutic potential found in the plant kingdom, ranging from the mechanistic analysis of ferroptosis and PI3K/Akt modulation to the clinical validation of traditional formulas in reducing chemotherapy-induced neuropathy. A vital step in increasing treatment effectiveness and improving patient satisfaction is the combination of chemotherapy with plant-derived bioactives. A more efficient, individualized, and patient-centered future in cancer treatment is possible if the oncology community embraces rigorous science, promotes interdisciplinary approaches, and leverages the use of state-of-the-art computational tools.
